# Experimental capabilities for liquid jet samples at sub-MHz rates at the FXE Instrument at European XFEL

**DOI:** 10.1107/S1600577523008159

**Published:** 2023-10-20

**Authors:** F. A. Lima, F. Otte, M. Vakili, F. Ardana-Lamas, M. Biednov, F. Dall’Antonia, P. Frankenberger, W. Gawelda, L. Gelisio, H. Han, X. Huang, Y. Jiang, M. Kloos, T. Kluyver, M. Knoll, K. Kubicek, I. J. Bermudez Macias, J. Schulz, O. Turkot, Y. Uemura, J. Valerio, H. Wang, H. Yousef, P. Zalden, D. Khakhulin, C. Bressler, C. Milne

**Affiliations:** a European XFEL, Holzkoppel 4, 22869 Schenefeld, Germany; b Fakultät für Physik, Technical University Dortmund, Dortmund, Germany; c Center for Free-Electron Laser Science CFEL, Deutsches Elektronen-Synchrotron DESY, Notkestr. 85, 22607 Hamburg, Germany; d Faculty of Physics, Adam Mickiewicz University, 61-614 Poznań, Poland; e The Hamburg Centre for Ultrafast Imaging, 22761 Hamburg, Germany; f Institut für Experimentalphysik, Universität Hamburg, 22607 Hamburg, Germany; ESRF – The European Synchrotron, France

**Keywords:** X-ray free-electron laser, XES, WAXS, XRD, utrafast science, pump–probe, liquid jets

## Abstract

The Femtosecond X-ray Experiments (FXE) instrument at the European X-ray Free-Electron Laser (EuXFEL) provides an optimized platform for investigations of ultrafast physical, chemical and biological processes. It operates in the energy range 4.7–20 keV accommodating flexible and versatile environments for a wide range of samples using diverse ultrafast X-ray spectroscopic, scattering and diffraction techniques. FXE is particularly suitable for experiments taking advantage of the sub-MHz repetition rates provided by the EuXFEL. In this paper a dedicated setup for studies on ultrafast biological and chemical dynamics in solution phase at sub-MHz rates at FXE is presented. Particular emphasis on the different liquid jet sample delivery options and their performance is given. Our portfolio of high-speed jets compatible with sub-MHz experiments includes cylindrical jets, gas dynamic virtual nozzles and flat jets. The capability to perform multi-color X-ray emission spectroscopy (XES) experiments is illustrated by a set of measurements using the dispersive X-ray spectrometer in von Hamos geometry. Static XES data collected using a multi-crystal scanning Johann-type spectrometer are also presented. A few examples of experimental results on ultrafast time-resolved X-ray emission spectroscopy and wide-angle X-ray scattering at sub-MHz pulse repetition rates are given.

## Abstract 2

A platform for experiments investigating the ultrafast dynamics of biological and chemical systems in solution phase at sub-MHz rates at the FXE Instrument at European XFEL is presented. Examples of fs-resolved X-ray emission spectroscopy and wide-angle X-ray scattering on typical spin-crossovers are given.

## 1. Introduction

The inauguration of the European X-ray Free-Electron Laser (EuXFEL) facility in 2017 marked the culmination of several breakthroughs in the hard X-ray free-electron laser sciences, most notably the application of superconducting accelerator technology allowing a high electron energy of up to 17.5 GeV and a 4.5 MHz repetition rate X-ray pulse pattern (Decking *et al.*, 2020 ▸; Abeghyan *et al.*, 2019 ▸; Sinn *et al.*, 2019 ▸). Currently, the EuXFEL operates three SASE beamlines that feed seven independent instruments (Mancuso *et al.*, 2019 ▸; Galler *et al.*, 2019 ▸; Meyer *et al.*, 2020 ▸; Madsen *et al.*, 2021 ▸; Zastrau *et al.*, 2021 ▸; Grychtol *et al.*, 2022 ▸; Carley *et al.*, 2022 ▸). The superconducting linear accelerator used in the EuXFEL provides free-electron laser (FEL) radiation in a 10 Hz burst mode, with each burst (or train) containing up to 2700 individual pulses at an intra-train rate of 4.5 MHz (Decking *et al.*, 2020 ▸). The total number of X-ray pulses in each train can be changed in a flexible manner to accommodate the needs of each experiment. This allows, for example, very high average photon flux or the use of single, on-demand X-ray flashes.

The high brilliance and high repetition rate of EuXFEL sets the groundwork for the investigation of chemical dynamics in very dilute samples and/or those not available in large quantities (*e.g.* proteins, samples presenting low solubility or difficult to synthesize) and enables new photon-demanding experimental techniques, *e.g.* valence-to-core X-ray emission spectroscopy (VtC XES), resonant X-ray emission spectroscopy, inelastic X-ray scattering, X-ray Raman scattering, among others (Lancaster *et al.*, 2011 ▸; March *et al.*, 2015 ▸, 2017 ▸; Katayama *et al.*, 2013 ▸; Britz *et al.*, 2020 ▸; Kern *et al.*, 2013 ▸, 2014 ▸; Szlachetko *et al.*, 2014 ▸, 2017 ▸; Kayser *et al.*, 2019 ▸; Fuller *et al.*, 2021 ▸). A prominent research field at FEL facilities is the investigation of the dynamics of chemical processes on the pico- and femto-second timescale (Zhang & Gaffney, 2015 ▸; Bergmann *et al.*, 2021 ▸). Such experiments are commonly carried out via the pump–probe method, through optical excitation (pump) and X-ray probe, on liquid samples (Zhang *et al.*, 2014 ▸; Canton *et al.*, 2015 ▸; Lemke *et al.*, 2017 ▸; Kunnus *et al.*, 2020 ▸; Bergmann *et al.*, 2021 ▸; Kinschel *et al.*, 2020 ▸; Bacellar *et al.*, 2020 ▸; Bacellar *et al.*, 2023 ▸; Canton *et al.*, 2023 ▸; Sension *et al.*, 2023 ▸).

The Femtosecond X-ray Experiments (FXE) instrument at the EuXFEL offers the possibility to probe liquid samples with hard X-rays during ultrafast pump–probe studies. A detailed description of the baseline instrumentation and its performance, in particular for studies of chemical dynamics in the liquid phase, has already been described elsewhere and thus will not be repeated here (Galler *et al.*, 2019 ▸; Khakhulin *et al.*, 2020 ▸). In the following sections we will report on further improvements of experimental capabilities of FXE for studies of biological/chemical dynamics in solution at sub-MHz rates, in particular those enabled by the development of a new dedicated sample chamber. New developments on high-speed liquid jets and examples of static and ultrafast time-resolved wide-angle X-ray scattering (WAXS), X-ray diffraction (XRD) and X-ray emission spectroscopy (XES) data collected using both dispersive and scanning multi-crystal X-ray spectrometers will also be given. The setup described here has already been successfully used for various experiments including recently published results (Bacellar *et al.*, 2023 ▸; Canton *et al.*, 2023 ▸; Sension *et al.*, 2023 ▸; Aleksich *et al.*, 2023 ▸).

## 2. FXE instrument overview

The FXE instrument is located at the SASE1 beamline of the EuXFEL, which currently provides FEL radiation for two instruments (Abeghyan *et al.*, 2019 ▸; Sinn *et al.*, 2019 ▸). It operates in the range 4.7–20 keV and was engineered to have a spacious, in-air sample interaction area capable of adopting various sample environments for flexible setups (Galler *et al.*, 2019 ▸). To maximize the use of space around the sample, FXE is equipped with a Stäubli TX-90L six-axis robot arm, mounted on a support tower above the interaction region. The flexibility of the robot arm allows several detectors used in XES, XRD and other experiments to be freely positioned. On the tower, perpendicular to the beam propagation direction, there is an additional manual encoded translation stage used for larger movements of the robot arm (total range 1.3 m) for increased detection capabilities. The whole system is mechanically designed to ensure detector positioning precision better than 50 µm, *i.e.* the smallest detector pixel size currently available at the instrument.

Several different X-ray detectors are available at FXE, either integrating over the entire burst (10 Hz operation) or capable of MHz rates detection, thus allowing for pulse-resolved data acquisition. Avalanche photo-diodes (APDs) and positive intrinsic negative (PIN) diodes are available as point detectors, as those provide decay times fast enough to resolve the individual pulses at 4.5 MHz repetition rates (Baron *et al.*, 2006 ▸; Owen *et al.*, 2009 ▸). A series of APD and PIN diodes are installed throughout FXE serving as incoming intensity (*I*_0_) monitors. For instance, an intensity and position monitor has previously been described by Galler *et al.* (2019 ▸). Additional *I*_0_ monitors include a set of four PIN diodes picking up scattering from the diamond window separating the optics branch from the experimental area and another PIN diode detecting scattering from the helium in the flight tube after the diamond window. Alternatively, a thin polyimide foil can be inserted in the beam path to increase the scattering intensity on this diode on experiments using weak beams (*e.g.* during operation with monochromatic X-rays). One extra PIN diode is also placed right at the entrance of the sample chamber described here. Gotthard-I and Gotthard-II 1D detectors (Mozzanica *et al.*, 2012 ▸; Zhang *et al.*, 2021 ▸) are available, as well as two JUNGFRAU (Redford *et al.*, 2018 ▸) and one Photon-III (Bruker) for 2D spatially resolved detection. Notably, a specifically developed multiple-gain stage detector, LPD (Koch *et al.*, 2013 ▸; Wheater *et al.*, 2022 ▸), allows for MHz-resolved scattering and diffraction experiments. An X-ray gas monitor (XGMD) was recently installed downstream of the sample interaction area providing pulse-resolved information about the absolute X-ray intensity at the sample position (Grünert *et al.*, 2019 ▸). The XGMD is useful during beam alignment and optimization; however, it does not have enough sensitivity to be used as a detector for experiments.

For optical excitation of samples FXE relies on the EuXFEL high-power MHz pump–probe laser (PPL). This femtosecond laser system delivers a fundamental wavelength of 800 nm and is capable of following the unique burst mode of EuXFEL, providing pulses of 50 fs duration or alternatively also 15 fs (Palmer *et al.*, 2019 ▸). The radiation is routinely frequency converted to its second and third harmonics, providing femtosecond laser pulses with 400 and 267 nm wavelengths, respectively. Additional tunability across the UV–Vis–NIR spectrum is achieved by a recently installed commercial optical parametric amplification system (TOPAS Prime), which is currently set to operate at 0.282 MHz maximum intra-train repetition rate. The PPL typically provides around 800 µJ pulse^−1^ in the fundamental. It is then split with a retractable 80:20 (R:T) beamsplitter into the TOPAS and harmonic generation (xHG) branches, respectively. This enables experiments with two-color optical excitation. The TOPAS delivers >10 µJ pulse^−1^ in the ranges 450–1000 nm and 1160–2600 nm, ∼4 µJ pulse^−1^ between 320 nm and 390 nm, and ∼2 µJ pulse^−1^ between 250 nm and 300 nm. In this configuration the pulse energy in the second harmonic is typically 40 µJ pulse^−1^ and 10 µJ pulse^−1^ in the third harmonic.

Two multi-crystal high-energy-resolution X-ray spectrometers are installed at FXE on either side of the sample interaction area. The dispersive-type X-ray spectrometer [von Hamos spectrometer (VHS)], inspired by a previously reported design (Alonso-Mori *et al.*, 2012 ▸), operates in von Hamos geometry (von Hámos, 1932 ▸) and can be equipped with up to 16 individual cylindrical crystal analyzers with 0.5 m radius of curvature. The other is a multi-crystal scanning-type spectrometer [Johann Scanning Spectrometer (JSS)] operating in Johann geometry employing up to five individually positioned spherical crystal analyzers of 1 m radius following the design by Bergmann & Cramer (1998 ▸) and used in several synchrotrons worldwide (Kleymenov *et al.*, 2011 ▸; Sokaras *et al.*, 2013 ▸; Kvashnina & Scheinost, 2016 ▸; Glatzel *et al.*, 2021 ▸). Both spectrometers operate in the vertical dispersion geometry.

A heavy-duty ten-axis sample manipulator stage (SMS) from Huber is used as the main support for a dedicated sample environment. The SMS is placed about 230 mm below the X-ray beam and has a horizontal flat area of 45 cm × 45 cm with a breadboard allowing placement and alignment of different equipment, *e.g.* a chamber for liquid jet experiments (see Section 3), a four-axis kappa goniometer, a chamber for solid-state diffraction experiments, cryostats, *etc*. Moreover, both X-ray spectrometers are mechanically connected to the SMS and can be rotated around their vertical axis for experiments exploring angular dependence. The whole SMS, including both VHS and JSS spectrometers, the LPD detector and the post-diagnostic table, is equipped with airpads and can be freely positioned anywhere inside the FXE experimental hutch. All experiments involving liquid samples are currently performed using a dedicated chamber, which will be described in detail in the next section.

## 3. Sample chamber for experiments using liquid jets

**Figure 1:**
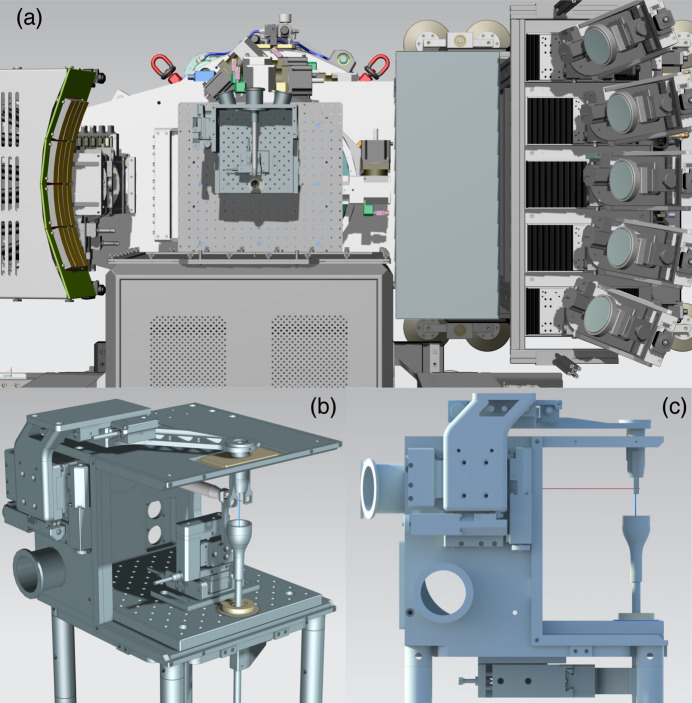
Top view of the sample interaction area at FXE (*a*) showing the dedicated chamber for experiments in liquid phase using high-speed jets, the VHS and JSS spectrometers on either side of the chamber and the LPD at a nominal distance of 200 mm from the liquid jet. 3D (*b*) and side (*c*) views showing the large opening allowing for simultaneous experiments to be performed, *e.g.* XAS, XES, XRD and WAXS.

The spacious and flexible sample interaction region at FXE combined with the high photon flux of EuXFEL is particularly beneficial to experiments using liquid samples. Due to the MHz repetition rates and the requirement to investigate samples in various solvents, often under low oxygen-exposure or at very low concentrations, a dedicated sample chamber was designed. Additional to providing a flexible and robust platform for liquid chemistry experiments at FXE, key design factors were the incorporation of large windows allowing simultaneous spectroscopic and diffraction/scattering experiments using both spectrometers and the LPD detector, respectively, as well as providing easy access to the jet components for manipulation and maintenance. The chamber also provides enough internal space for placing additional equipment for X-ray and laser beams conditioning and diagnostics, as well as for jet visualization. Figure 1 ▸ shows CAD-renderings of the chamber design and illustrates the large opening angles of 264° in the horizontal and 133° forward vertical directions around the liquid jet. Placing the LPD detector at a typical distance of about 150 mm from the liquid jet allows reaching up to 8 Å^−1^ at 12 keV and 11 Å^−1^ at 16.5 keV. The high photon energy and short sample–detector distance results in improved nominal structural determination in time-resolved WAXS and XRD experiments in comparison with typically reported results on liquid-phase photo-chemistry experiments at XFELs (Canton *et al.*, 2015 ▸; van Driel *et al.*, 2016 ▸; Kunnus *et al.*, 2020 ▸; Heo *et al.*, 2022 ▸; Reinhard *et al.*, 2023 ▸; Katayama *et al.*, 2023 ▸).

The chamber interior can be completely enclosed with a window (*e.g.* Kapton or Mylar) supported on a frame attached by magnets. Three feedthroughs with SMA and motor connectors are available for internal sensors or detectors (*e.g.* *I*_0_, total fluorescence signal, *etc*.) and actuators. The entire chamber is made from aluminium (CNC machined) and has a modular design, allowing for easy replacement and/or adjustments of specific aspects of the design. Three precision motors (Huber Linear Stage 5101.07) are used to position the jet along the three Cartesian coordinates with a 1 µm reproducibility. These motors are mounted outside the chamber to protect them from possible contamination due to spraying or leaks in the liquid jets. The wide jet support placed outside the chamber allows for different nozzle designs to be easily mounted in a reproducible way. With this design, further nozzle changes or upgrades can be accommodated without the need to redesign the whole jet support. At the bottom of the chamber a sample catcher feeds into a 5 mm-diameter tube, guiding the jet back to the sample reservoir placed below. This catcher tube can be precisely adjusted by two perpendicular OWIS VT30 manual stages. Importantly, this minimizes spraying since direct surface contact of the high-speed jet can be reduced. The chamber is installed on the SMS without the top two swivel stages, leaving a large free space of 225 mm underneath it to place the sample reservoir or any additional equipment needed. A seal for the openings through which the jet and catcher extend into the chamber is achieved by two pairs of sliding plates made of polytetrafluoroethylene (PTFE) and held by small magnets. These weak magnets maintaining contact between the PTFE sliding plates do not affect the accuracy of the jet positioning. Two KF-40 flanges placed at the upstream side of the chamber are available either as viewports or to allow passage for the pump laser at an angle of about 15°. Alternatively, quasi-collinear laser and X-ray propagation is also possible by in-coupling the laser upstream of the chamber. A third rectangular viewport is placed at the top of the chamber. A detachable breadboard with M6-threaded holes is mounted at the base of the chamber, allowing easy mounting of small equipment inside the chamber. A gas inlet enables placing the sample in different atmospheres. Helium is most commonly used as the filling gas to minimize signal attenuation and spurious scattering. A 2 mm-thick silver cylinder is used as flight tube, containing the beam up to about 15 mm before the jet. Two additional motorized stages can also be used to position a pinhole or a fixed-size slit right before the sample. A helium level detector (New Cosmos Portable XP-3140-ATEX) can also be placed in the vicinity of the liquid jet. Custom-made trapezoidal-shaped flight paths filled with helium can be placed between the chamber windows and the spectrometer in order to further minimize signal attenuation by air.

## 4. High-speed liquid jet systems

Sample delivery for MHz pump–probe X-ray experiments has to ensure that the probed sample volume is replenished rapidly after each probe pulse. The enormous energy of a single ultrashort X-ray pulse (∼2.5 mJ at SASE1) focused to a few µm spot causes the irradiated segment of the jet to be vaporized, leaving behind gaps in the liquid column (Stan *et al.*, 2016 ▸; Vagovič *et al.*, 2019 ▸). Moreover, each explosion generates a shock wave that propagates both upstream and downstream, possibly disturbing the sample volume probed by the subsequent pulses (Stan *et al.*, 2016 ▸; Vagovič *et al.*, 2019 ▸). Out of the many existing methods, high-speed liquid microjets can deliver samples at sufficient velocities to use the pulse rates at EuXFEL (Grünbein *et al.*, 2021 ▸; Vakili *et al.*, 2022 ▸). The different liquid microjets currently used at FXE are detailed below.

Ultrafast hard X-ray spectroscopy experiments in dilute solutions usually require rather large jet diameters (20–200 µm) due to the different absorption cross-sections of the optical laser and X-rays and the need to balance transient signal magnitude and the temporal resolution. The latter is a combination of both laser and X-ray pulse duration, the relative laser/X-ray pulse arrival time jitter and the group velocity mismatch resulting in a temporal walk-off of the two propagating pulses (typically by ∼1 fs µm^−1^). Other factors to be taken into account when optimizing the pump–probe signal are the maximum sample solubility and available quantity. A compromise is often achieved with jets with diameters in the range 50–100 µm, with a few experiments using 30–50 µm jets when higher temporal resolution is required to resolve the fastest processes and the samples exhibit high solubility and/or large optical absorption (Lemke *et al.*, 2017 ▸; Katayama *et al.*, 2019 ▸).

The majority of liquid-phase pump–probe experiments currently performed at FXE employ high-speed Rayleigh jets in order to profit from the high pulse repetition rates (Galler *et al.*, 2019 ▸; Khakhulin *et al.*, 2020 ▸). In this regard, glass-based nozzles (Advanced Microfluidic Systems GmbH – AdMiSys) coupled with large-scale preparative solvent delivery units (Shimadzu LC20-AP Prominence) have proved to be a viable solution to produce cylindrical Rayleigh jets with diameters in the range 75–150 µm and velocities of up to 60 m s^−1^. However, these commercial glass-based nozzles have limitations and drawbacks such as high cost, etching/delivery times and lack of design varieties. Robust and flexible nozzle designs can nowadays be achieved by 3D printers based on the two-photon polymerization technique (Knoška *et al.*, 2020 ▸; Vakili *et al.*, 2022 ▸).

### 4.1. Nozzle design choices and assembly

Tailored 3D printed jet devices can be fabricated in-house with highly reproducible geometric features, are cost-efficient and promise nearly unlimited design flexibility with rapid and convenient implementation of geometric variations. In a recent example, modification of the conventional design for the gas dynamic virtual nozzle (GDVN) (DePonte *et al.*, 2008 ▸), which is routinely used for serial femtosecond crystallography (SFX) experiments, allowed for the implementation of wider liquid orifices (*e.g.* 150 µm) generating gas-focused liquid jets with diameters in the range 7–50 µm. Those jets are unfortunately too slow (*v* ≤ 25 m s^−1^) for full MHz pulse repetition rates, but still suitable for experiments at 0.141 to 0.564 MHz rates providing relatively large jet diameters while consuming less total sample volume compared with the gas-less Rayleigh jets. For instance, a 10 µm-wide liquid jet can be operated at flow rates below 0.1 ml min^−1^ (using a GDVN with a 150 µm liquid orifice) and deliver samples efficiently for experiments at 0.564 MHz pulse operation mode. One promising application for such nozzles is small-molecule serial femtosecond X-ray crystallography (smSFX) experiments (Schriber *et al.*, 2022 ▸), which can also be performed at FXE by simple adaptation of the jet support to fit the GDVN (Aleksich *et al.*, 2023 ▸). These GDVN jets operate in a non-recirculating manner using typically 5 ml of sample loaded on a piston, each lasting for about 1 h.

**Figure 2:**
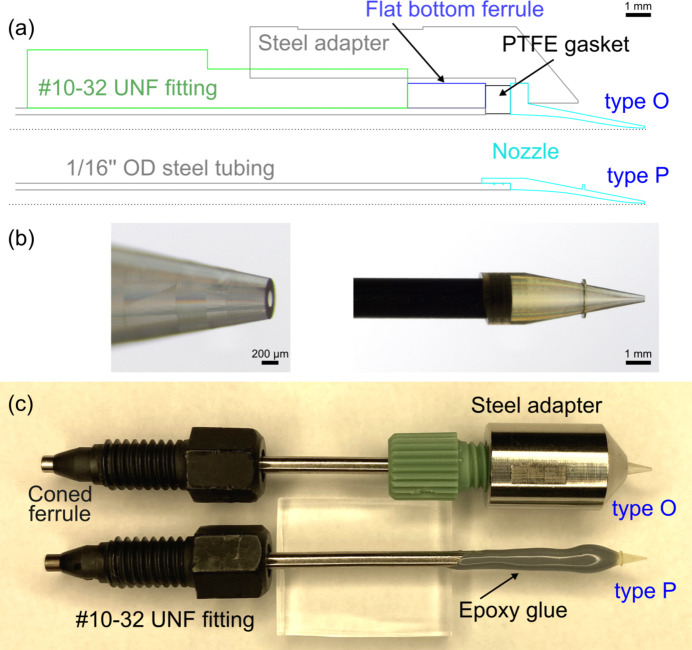
(*a*) Schematic representation of the 3D printed nozzle assemblies. Screwed version (type O) (top) and glued version (type P) (bottom). (*b*) Close-ups of the type P nozzle (100 µm inner diameter) prior to gluing and (*c*) side-by-side picture of both assemblies. The length of the steel tubing is 50 mm.

Inspired by the proven AdMiSys glass tips, two 3D printed nozzle designs [conical tubing tips (CTTs)] were developed [Fig. 2 ▸, panels (*a*) and (*c*)]. In the first design (type P), the 3D printed nozzle (channel length: 5.35 mm; total length: 6.5 mm) contained a port allowing the insertion of a 1/16 inch outer-diameter steel tubing (IDEX, U-145) which was glued using epoxy adhesive (Loctite 3450). The tubing inner diameter was 0.046 inch, matching the internal diameter of the nozzle at the steel-polymer interface (1.19 mm). Further downstream, the nozzle interior is tapered to facilitate a 100 µm-wide opening at the tip [panel (*b*) in Fig. 2 ▸]. Other variants offer a tapering down to either 50 or 25 µm at the tip. After gluing, the steel-nozzle assembly was cured for 12 h at 80°C.

In a second design variation (type O), the sealing was achieved without any epoxy-based glue, which might degrade when exposed to certain organic solvents. To ensure leak-free operation with organic solvents, which are commonly used in the liquid phase pump–probe experiments at FXE, the assembly was composed of a PEEK fitting with an external #10-32 UNF thread (IDEX, M-653) in combination with a flat-bottomed ferrule (IDEX, M-250). The fitting contained a through-hole for 1/16 inch outer-diameter tubing for the connection to the liquid line. The nozzle also provides a flat bottom with a base external diameter matching the width of the ferrule (3.68 mm). Between the ferrule and the nozzle, a thin PTFE washer was inserted. These flexible gaskets with an outer diameter of 3.5 mm and a center through-hole of 1.25 mm in diameter were cut out of 1.0 mm-thick PTFE sheets (COG). Sealing was then achieved by pressing the fitting into a custom steel adapter with an internal #10-32 UNF thread and a through-hole slot for the nozzle. Further details on nozzle fabrication (instrumentation, slicing, printing, development of the 3D printed devices) can be found elsewhere (Vakili *et al.*, 2022 ▸). An online repository of 3D designs for high-speed liquid sample delivery (nozzles, as well as adapters and beamline connection parts) can be found at https://github.com/flmiot/EuXFEL-designs.

An important advantage of 3D printed nozzles is the possibility of conveniently exploring alternative nozzle tip designs and hence jet shapes. In this regard, liquid jets with truly flat-surface profiles offer several advantages with respect to the cylindrical shape of conventional Rayleigh jets. A flat-surface jet eliminates a lensing effect which would otherwise arise from refraction of the optical light at curved surfaces and could result in uneven excitation (Galinis *et al.*, 2017 ▸; Crissman *et al.*, 2022 ▸). Furthermore, the possibility to explore the extended flat and uniform liquid surface on, for example, hard X-rays transient grating or dispersive X-ray absorption spectroscopy (XAS) experiments is additionally enabled (Rouxel *et al.*, 2021 ▸; Katayama *et al.*, 2013 ▸; Obara *et al.*, 2014 ▸). Several works describing the generation of sheet-like liquid jets utilizing either colliding jets (Ekimova *et al.*, 2015 ▸) or compressed-gas-driven jets (Crissman *et al.*, 2022 ▸) have been recently reported. Modern 3D printing based on two-photon polymerization has also been exploited for the fabrication of such devices (Galinis *et al.*, 2017 ▸); however, sheets with sub-micrometre thickness have mostly been the core of those developments and are not appropriate for the hard X-ray pump–probe experiments at FXE. Using the design flexibility provided by our 3D printed approach, we have modified our original type-O CTT design to feature apertures with a rectangular cross-section at the nozzle tip to generate Rayleigh jets with moderately flat jet areas. The dimensions of each region are determined by the smaller axis of the slit geometry and this approach was used to produce flat jets with thicknesses varying in the range 10–30 µm.

### 4.2. Nozzle operation and jetting capabilities

**Figure 3:**
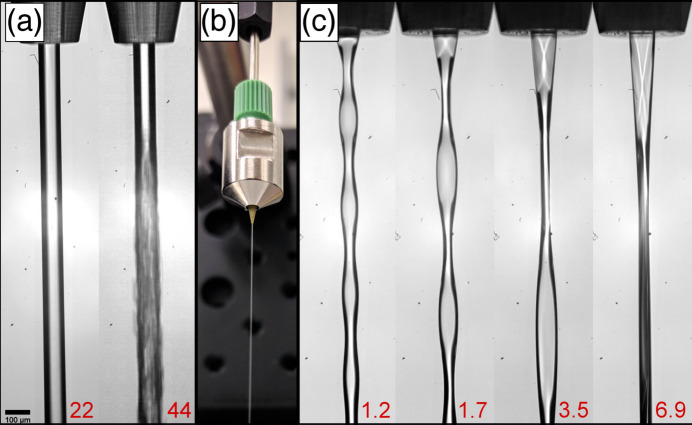
(*a*) Images of O-type CTT nozzles pumping water at (left) 22 ml min^−1^ and (right) 44 ml min^−1^, resulting in 100 µm-wide cylindrical liquid jets at atmospheric conditions. (*b*) Photograph of the O-type nozzle (inner diameter = 100 µm) during operation. (*c*) Rectangular slit nozzle with a 30 µm × 100 µm cross-section in operation with varying liquid flow rate. The numbers in the bottom right (1.2, 1.7, 3.5, 6.9) denote the applied liquid flow rate in ml min^−1^ and achieve the sample velocities required for operation at 0.141, 0.282, 0.565 and 1.13 MHz, respectively.

The AdMiSys glass nozzles (outer diameter = 3 mm, length = 20 mm) provide an inner diameter of 1 mm at the beginning, which tapers down to 30–100 µm at the tip. In order to produce stable jets with sufficient speeds compatible with the EuXFEL MHz operation, an extra connective PEEK tubing with a sufficiently large inner diameter (*e.g.* outer diameter of 0.03 inch, inner diameter of 250 µm) inserted into the glass piece all the way down to the tapering at the tip is required. Since there are no glued parts in the AdMiSys nozzles, they provide optimum chemical compatibility with most solvents used in liquid chemistry experiments at FXE, *e.g.* water, acetonitrile, DMF, DCM, ethanol, 2-propanol, toluene, *etc*. In this configuration, the AdMiSys nozzles produce stable liquid jets when using flow rates in the range 1–30 ml min^−1^ (see Fig. 3 ▸), resulting in a calculated flow velocity of about 64 m s^−1^ for a 100 µm-diameter nozzle at the highest flow rate. A jet speed of about 113 m s^−1^ is needed to refresh a 100 µm-long jet region for 1.1 MHz operation and, equivalently, about 56 m s^−1^ are needed for 0.564 MHz operation. The highest jet speed obtained for 100 µm AdMiSys nozzles is compatible with operation at 0.564 MHz, neglecting the secondary effects of the up- and down-stream shock wave previously reported by Stan *et al.* (2016 ▸) and Engel *et al.* (2021 ▸).

The P-type CTTs use epoxy adhesive in the connections; hence, the chemical compatibility with different solvents needs to be carefully checked for each experiment. The nozzle material itself was proven to be compatible with a large set of solvents.^1^ Tests with water indicate a similar performance as the AdMiSys nozzles, *i.e.* stable jets compatible with 0.564 MHz operation using about 30 ml min^−1^ flow rates. The more chemically robust O-type CTTs were also tested with water and provided similar performance as the AdMiSys nozzles and the P-type CTTs with equivalent orifices. Figure 3 ▸(*a*) shows microscopic images of the 100 µm-diameter O-type CTTs during tests with water. On the left the flow rate was set to 22 ml min^−1^, and on the right it was increased to 44 ml min^−1^, resulting in a theoretical flow velocity of approximately 47 m s^−1^ and 90 m s^−1^, respectively. Though the latter condition seems encouraging for pump–probe experiments at 1.1 MHz, an inspection of high-speed microscopy images at short exposure times revealed a highly turbulent flow in the vicinity of the tip. Nozzles with smaller inner diameters allow the use of significantly lower flow rates to generate laminar-flowing high-speed jets. For instance, in order to refresh a 100 µm footprint at 0.564 MHz and 1.1 MHz, a CTT with 50 µm-wide aperture requires merely 6.6 ml min^−1^ to achieve a sample velocity of about 56 m s^−1^ and 13.3 ml min^−1^ to provide a 113 m s^−1^ fast jet, respectively.

Tests with jetting water on a prototype rectangular nozzle tip having 30 µm × 100 µm cross-section indicated relatively stable operation, with minor oscillations of near sheet-like areas, as shown in Fig. 3 ▸(*c*). They presented varying lengths and distances between ‘flipping zones’ upon increasing flow rates. A stable flow with a flat jet area of 100 µm width and 30 µm thickness was reached with a flow of 6.9 ml min^−1^. Further development on flat jets suitable for experiments at FXE is ongoing.

Independent from the specific design, both cylindrical and rectangular jets described here operate using the same pump system, requiring a minimum of 25 ml of solution for the recirculating flow. A compilation of the relevant performance parameters of the different jet designs is presented in Table 1 ▸.

### 4.3. Droplet-on-demand jet

Some experiments using liquid jets cannot afford the large amounts of sample needed to operate the standard Rayleigh or GDVN jets. For instance, when the amount of microcrystals suspension sample in SFX experiments is limited to less than a couple of ml, GDVN jets cannot be used due to a relatively high sample flow rate and total volume needed (Fuller *et al.*, 2017 ▸; Miller *et al.*, 2019 ▸; Chatterjee *et al.*, 2019 ▸). Additionally, since the sample is not recovered after jetting, the continuous flow of the GDVN jets is not optimal for the burst mode operation of EuXFEL as most of the time the sample is not exposed to X-rays, resulting in a poor measurement duty-cycle and wasted sample. Droplet-on-demand (DoD) injection is a promising solution to reduce the sample consumption in SFX experiments. In experiments using DoD, the liquid jet sample is delivered as droplets synchronized to the X-ray pulse trains using a pneumatic microdispensing system based on a pressurized reservoir and a piezoelectric actuator. That allows not only a much smaller sample consumption but also using nozzles of larger diameter and more concentrated suspensions, resulting in an increased crystal hit-rate. Microdispensing systems suitable for these applications are commercially available and can easily be coupled to the nozzles and chamber presented here. Preliminary tests indicate that a DoD can produce up to nine droplets per train at 23.5 kHz. The use of DoD sample injection in SFX experiments at FXE will be the subject of a future publication.

## 5. X-ray emission spectroscopy

Non-resonant XES has been widely used to investigate ultrafast processes in liquid phase systems (Zhang *et al.*, 2014 ▸; Mara *et al.*, 2017 ▸; Zhang *et al.*, 2017 ▸; Kunnus *et al.*, 2020 ▸; Naumova *et al.*, 2020*a* ▸,*b* ▸; Smolentsev *et al.*, 2020 ▸). The majority of those studies were performed using dispersive high-resolution X-ray spectrometers operating in von Hamos geometry. The dispersive geometry has the advantage of collecting the full emission spectrum at once per shot, minimizing the issues related to the incoming pulse intensity fluctuations typical of SASE operation. The multi-analyzer von Hamos spectrometer (VHS) at FXE can be equipped with up to 16 (500 mm-radius) cylindrical crystal analyzers operating in Bragg angles ranging from 65° up to about 84° and has been described elsewhere (Galler *et al.*, 2019 ▸). The 500 mm working distance of the VHS at FXE was chosen to provide a good compromise between energy resolution and collection efficiency, while maintaining the possibility to collect multiple emission lines simultaneously by either using several different analyzers or by slightly changing the Bragg angles. Naturally, the final energy resolution of dispersive X-ray spectrometers is also a function of the beam size along the dispersive direction, the detector pixel size, the specific material of the analyzers and the Bragg angle of operation (Szlachetko *et al.*, 2012 ▸; Alonso-Mori *et al.*, 2012 ▸; Sahle *et al.*, 2023 ▸).

Simultaneous detection of multiple X-ray emission lines in a single experiment has become an attractive feature at FXE, taking advantage of the flexibility of the VHS and the availability of multiple sets of analyzer crystals, as also reported earlier (Kalinko *et al.*, 2020 ▸). The collection of multiple X-ray fluorescence channels simultaneously, *e.g.* the spin–orbit dominated *K*α and *p*–*d* exchange modulated *K*β X-ray emission lines in transition metals, enables extraction of additional element-specific chemical information. Furthermore, in cases in which weaker XES features are the focus of the investigation, an additional strong X-ray emission line can be collected serving as a more reliable internal normalization and aiding in the optimization of experimental conditions. Differences in the temporal response of various X-ray emission lines to the optical pump are accessible as well. In the context of ultrafast XES experiments on dilute systems, multi-color experiments make better use of scarcely available sample and increase the efficiency of beam time use. Additionally, they enable simultaneous measurement of XES signals of different components in multi-metallic centers, also complementing XAS measurements (Gul *et al.*, 2015 ▸; Canton *et al.*, 2015 ▸; Alonso-Mori *et al.*, 2016 ▸; Martinie *et al.*, 2018 ▸; Cammarata *et al.*, 2021 ▸; Liekhus-Schmaltz *et al.*, 2022 ▸). This trend is also being applied in other facilities, with interesting recent developments in small-radii analyzers fabrication with alternating strips of different crystal orientations (Rani *et al.*, 2020 ▸).

**Figure 4:**
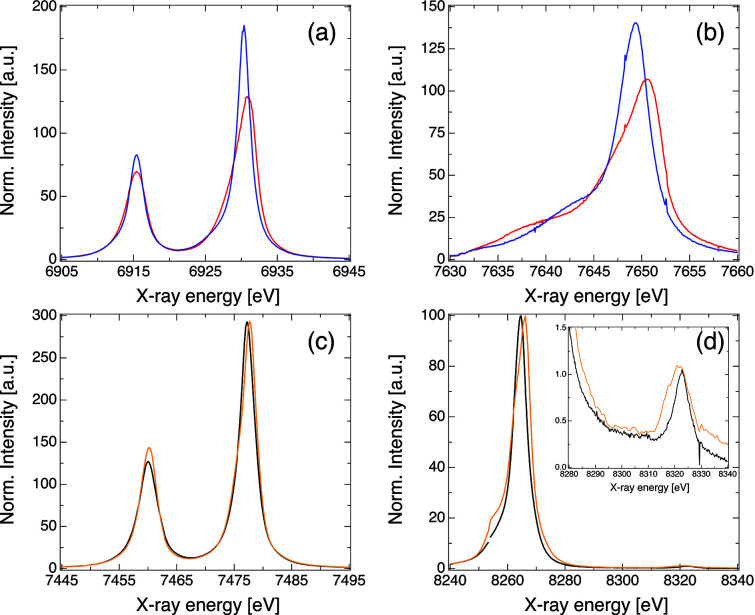
Examples of *K*α, *K*β and VtC XES measured simultaneously with the von Hamos spectrometer at FXE. The *K*α (*a*) and *K*β (*b*) XES of 5 m*M* solutions of [Co(bpy)_3_]^3+^ (blue) and [Co(bpy)_3_]^2+^ (red) in acetonitrile are shown. The limited dispersion window in the Co *K*β did not allow detecting the VtC region. The *K*α and *K*β/VtC of a Ni foil (black) and a 10 m*M* aqueous solution of Ni^II^(SO_4_) (orange) are shown in panels (*c*) and (*d*), respectively. The inset in panel (*d*) highlights the VtC region. The small discontinuity present in these data arises from the emission signal impinging in between two ASIC chips on the JUNGFRAU detector.

A set of multi-color XES data collected on selected nickel and cobalt compounds in both solid form and solution is shown in Fig. 4 ▸. Those included a Ni foil (6 µm), a 10 m*M* aqueous solution of Ni^II^ sulfate hexahydrate and 5 m*M* solutions of [Co(bpy)_3_]^2+^ and [Co(bpy)_3_]^3+^ (bpy = 2,2′-bipyridine) in acetonitrile. All samples were purchased from Dyenamo, Sigma-Aldrich or EXAFS Materials and were used without further purification. In these measurements the Ni foil was placed at 45° with respect to the incoming X-ray beam and one pulse per train (10 Hz) at 9.3 keV was used. Further measurements using ten pulses per train at 0.564 MHz did not indicate noticeable distortion effects in the spectrum. The solutions were delivered by a 100 µm-thick AdMiSys jet flowing at approximately 30 ml min^−1^. The incoming X-rays were set to 9.3 keV (SASE bandwidth) with a total of 150–180 pulses train^−1^ at 0.564 MHz, which was sufficient to ensure complete replenish of the probed volume between consecutive X-ray pulses. In all cases the incoming pulse energy varied between 1 and 3 mJ at the source and a He flight path between the sample–analyzers–detector was used to minimize the signal attenuation by air. The VHS was equipped with seven Si(531) analyzers positioned at about 64.6° to disperse the Ni *K*α and eight Ge(111) at 66.7° to analyze the Ni *K*β and VtC. Since the Bragg angles for both emission lines were similar, they were detected by a single JUNGFRAU detector operating at a 10 Hz frame rate, *i.e.* integrating all pulses in a train per exposure. For the Co XES measurements the VHS was equipped with seven Si(531) analyzers at approximately 77° and seven Ge(111) analyzers at about 83° to analyze the Co *K*α and *K*β emission, respectively. The Bragg angles used for the cobalt measurements result in a large spatial separation between the Co *K*α and *K*β, hence two separate JUNGFRAU detectors were used, both operating at 10 Hz. The XES data presented here were normalized to their area.

The data shown in Fig. 4 ▸ illustrate the setup flexibility and the excellent data quality provided by the VHS at FXE. Under regular operation conditions (*i.e.* 9.3 keV, 2–3 mJ pulse^−1^ at the source and 100–150 pulses at 0.564 MHz repetition rates) we are able to measure the weak VtC signals from dilute solutions in about 10 min. The overall VHS energy resolution was evaluated by measurements of the elastic scattering in the configuration using the largest Bragg angle to minimize the geometric contributions in the resolution. The SASE beam energy was tuned to the vicinity of the cobalt *K*α_1_ (6930 eV) and *K*β (7649 eV), monochromated by a four-bounce Si(111) monochromator and scanned in steps of 10 eV. The elastic scattering from an aluminium target was modeled by a Gaussian and used in the pixel-to-energy spectra calibration. This resulted in a mean FWHM of 0.77 eV for *K*α and 0.87 eV for *K*β, attesting to the sub-eV resolution of the VHS.

**Figure 5:**
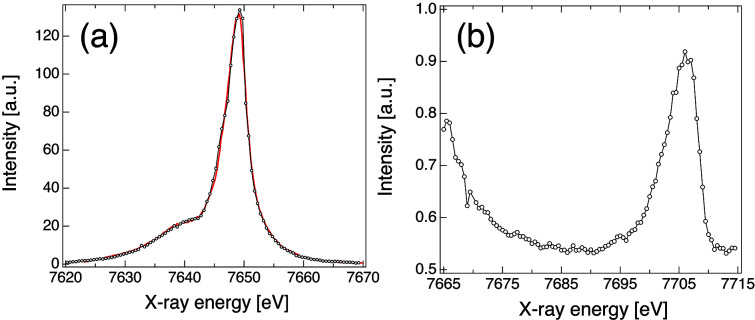
Example of *K*β (*a*) and VtC (*b*) XES of a cobalt metal foil measured with the Johann spectrometer at FXE. A reference Co foil *K*β measurement taken at a synchrotron (red) with a dispersive spectrometer is also shown in (*a*).

Another way to measure high-resolution XES data is to employ a scanning-type spectrometer operating in Johann geometry (Bergmann & Cramer, 1998 ▸). For this, FXE is also equipped with a five-analyzer JSS similar to others installed at several synchrotron facilities (Kleymenov *et al.*, 2011 ▸; Sokaras *et al.*, 2013 ▸; Kvashnina & Scheinost, 2016 ▸; Glatzel *et al.*, 2021 ▸). Here we are only presenting the first commissioning results, and further details on the JSS operation and performance will be the subject of a forthcoming publication. Figure 5 ▸ shows the *K*β and VtC XES from a metal foil collected with the JSS at FXE. In these measurements the SASE X-ray beam was centered at 9.3 keV with 5 pulses train^−1^ for the *K*β and 100 pulses train^−1^ for the VtC, always at 1.1 MHz. Two Ge(111) spherical analyzers with 1 m bending radius positioned at about 83° were used in the JSS to analyze the Co *K*β and VtC XES. The signal was detected by a JUNGFRAU detector which was held by the robot arm and followed the Rowland circle during the energy scans. A 0.5 eV step was used throughout the *K*β mainline and VtC regions, with 5 s acquisition per point. The XES data measured with the JSS shows comparable quality and energy resolution with the one measured with the VHS, with the advantage of concentrating all the signal in a single energy due to the Johann geometry. The use of the JSS will allow a more efficient collection of weak XES signals, as well as opening the possibilities for different spectroscopic measurements, *e.g.* high-energy-resolution fluorescence-detected XAS, X-ray Raman spectroscopy, *etc*.

## 6. X-ray diffraction

X-ray diffraction experiments on small molecules have recently been employed at XFELs to solve the structure of micro- and nano-crystals that proved unsuitable at synchrotrons (Schriber *et al.*, 2022 ▸). The methodology, called small-molecule femtosecond X-ray crystallography, relies on diffraction from microcrystalline suspensions supplied by a thin GDVN-based jet to obtain thousands of randomly oriented diffraction patterns which are subsequently indexed and used to solve the crystal structures using standard tools for single-crystal diffraction data.

**Figure 6:**
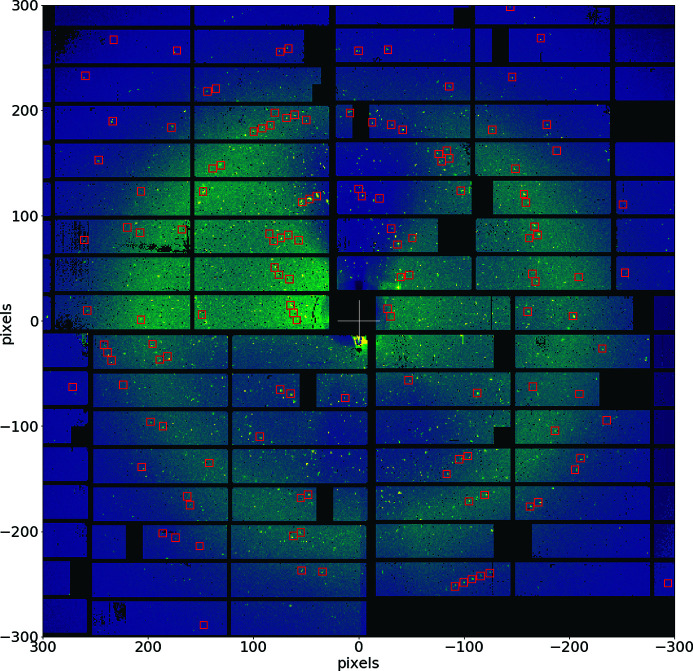
Typical single-shot diffraction pattern from lysozyme microcrystals delivered by the GDVN jets. Indexed reflections are marked with red squares. Despite a relatively low indexing rate of ∼1% in the dataset (with a total of 4.7 million frames) and the presence of frames with multiple crystal hits, the extracted unit cell is well defined and matches well the results reported in the literature (Weinert *et al.*, 2017 ▸).

These smSFX experiments and even regular SFX on proteins can be performed at FXE using the sample chamber and GDVN jets described above. The diffraction signals from randomly oriented crystals are detected on a shot-by-shot basis using the LPD detector. Alternatively, DoD injection can also be used in cases of limited sample availability. An example of a typical single-shot diffraction pattern collected with 9.3 keV X-rays from lysozyme microcrystals with 2–3 µm size distribution flowing through a 10 µm GDVN jet is shown in Fig. 6 ▸. The helium atmosphere inside the chamber was sufficient to reduce the scattering background to a level that it did not affect the peak finding and indexing procedures. The indexed reflections are highlighted by red squares in Fig. 6 ▸. During the data collection the direct beam was guided through the detector center in a silver tube placed directly at the chamber back window. Importantly, the sample–detector distance can be varied (usually in the range 150–500 mm) in order to collect high- and low-resolution crystallography data, improving the data completeness. Moreover, FXE allows exploring the advantages of high photon energies delivered by EuXFEL (up to 20 keV), which, combined with the large LPD detector area and the possibility of using short sample-detector distances, results in XRD data with nominal resolution down to about 0.8 Å. That should allow reaching atomic resolution on the structural determination via smSFX including the pump–probe configuration.

## 7. Time-resolved XES and WAXS capabilities

The sample chamber for experiments using liquid jets presented here supports different types of pump–probe experiments to be performed in the same experimental configuration in a quasi-simultaneous manner. However, the experimental conditions still need to be carefully adjusted in order to have optimum conditions for each measurement. WAXS signals are very sensitive to the liquid jet flow quality, which depend on many factors particular to each system under investigation: sample concentration, solvent viscosity, among others. Thus, pump–probe WAXS experiments at FXE are frequently realized at a reduced repetition rate compared with XES, while often attenuating the X-rays to prevent strong disturbances and ensuring a laminar liquid jet flow and detector linearity. On the other hand, XES experiments profit from an increased number of pulses at a higher repetition rate, in particular when detecting weak transient signals (*e.g.* low sample concentration, valence-to-core XES). Moreover, they are less affected by disturbances in the liquid jet flow caused by either laser or X-ray pulses. In the following, we show examples of pump–probe XES and WAXS experiments performed using the chamber described in this work. The experimental conditions for each experiment were optimized to obtain the best signal-to-noise ratio.

**Figure 7:**
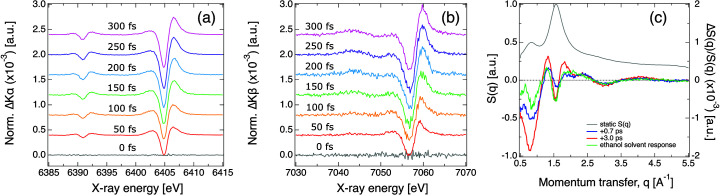
(*a*) Transient *K*α and (*b*) *K*β XES from a 45 m*M* aqueous solution of [Fe(terpy)_2_]^2+^ at several time delays after 400 nm excitation. Each time delay is shifted vertically for improved clarity. (*c*) Difference WAXS from a 15 m*M* solution of [Fe(bpy)_3_]^2+^ at 0.7 ps (blue) and 3 ps (red) time delays after 400 nm excitation. The green curve represents a reference ethanol solvent response from the literature (Kjaer *et al.*, 2013 ▸), while the gray curve is the static scattering from ethanol. Note the different scales on the *y*-axis.

As an example of the ultrafast pump–probe XES capabilities of FXE, we show in Fig. 7 ▸ the transient *K*α and *K*β XES of a 45 m*M* aqueous solution of [Fe(terpy)_2_]^2+^ (terpy = 2,2′:6′,2′′-terpyridine) upon 400 nm excitation. As a typical spin crossover model compound, [Fe(terpy)_2_]^2+^ undergoes a low-spin to high-spin transition after ultrafast photoexcitation of a metal-to-ligand charge transfer state, which is readily captured by characteristic *K*α and *K*β XES spectral changes (Vankó *et al.*, 2013 ▸, 2015 ▸; March *et al.*, 2017 ▸). The ultrafast pump–probe XES measurements shown here were performed using a similar configuration reported previously (Khakhulin *et al.*, 2020 ▸). Namely, the solution was flown by a 100 µm-diameter commercial Rayleigh jet (AdMiSys) at about 60 m s^−1^ and optically pumped with 400 nm, 50 fs pulses from the PPL operating at 5 Hz, *i.e.* pumping all X-ray pulses every second train, and 9 µJ pulse intensity. The sample concentration and optical spectrum were monitored on-the-fly by a UV–Vis spectrometer system (OceanOptics – OCEAN-FX-XR1-ES) flowing sample in a parallel closed circuit connected directly to the main sample reservoir. An additional pump (Shimadzu LC20-AT) was used to replenish the solvent lost during the experiment due to evaporation or spraying caused by the strong laser and X-ray pulses. Probing was done by the X-ray pulses with 9.3 keV incoming energy (SASE bandwidth), with 1.6 mJ pulse^−1^ at the source at 0.564 MHz intra-train repetition rate and a total of 88 pulses per train (effectively 880 pulses s^−1^). The laser and X-ray beam sizes were 70 and 10 µm FWHM, respectively. The XES spectra were measured using the VHS equipped with six Ge(110) crystals at about 75° and eight Si(531) crystals at 73° to select the iron *K*α (6403.8 eV) and *K*β (7058.0 eV), respectively. The signals were simultaneously collected by a JUNGFRAU detector integrating all pulses in every train. In this configuration, the experiments reach an instrument response function of about 115 fs without any pulse arrival time jitter correction (Khakhulin *et al.*, 2020 ▸).

The results of a typical femtosecond WAXS measurement on a 100 µm-thick liquid jet are shown in Fig. 7 ▸. An ethanol solution of [Fe(bpy)_3_]^2+^ (bpy = bipyridine) with concentration of 15 m*M* was flowed in the liquid jet system at 30 ml min^−1^ rate and was excited by 400 nm femtosecond pulses (70 fs FWHM duration). The radiation of 38 X-ray pulses per train with intra-train repetition rate of 141 kHz was used to collect pulse-resolved scattering with the LPD, which was centered on the beam and placed at a distance of 200 mm from the jet. The 12 keV X-ray beam with natural SASE bandwidth was focused to 10 µm size (FWHM) and was contained by a silver tube directly after exiting the sample chamber to reduce air scattering. In order to reduce the effect of jet perturbations by X-ray pulses and to avoid possible detector saturation artifacts, the incoming beam was additionally attenuated by a factor of three, resulting in an average pulse energy of 300 µJ on the sample. A sufficient amount of ground-state reference data were acquired by performing the optical excitation of the jet at 5 Hz train repetition rate with intra-train rate of 70.5 kHz, *i.e.* every other X-ray pulse in every second train was probing the optically excited sample. For every pump–probe delay setting the pulse-resolved scattering images from LPD were sorted according to the laser-on and laser-off conditions and azimuthally integrated to result in respective 1D WAXS curves. Those were then normalized to the total scattering intensity, averaged and used to produce the pump–probe difference (laser-on–laser-off) curves. Additionally, to compensate for potential artifacts from the laser-induced average heating of the jet with a train, a reference difference curve collected at a negative pump–probe delay of −1 ps was subtracted from each difference curve at a positive delay. The results obtained for pump–probe delays of 0.7 ps and 3 ps are shown in Fig. 7 ▸(*c*) (blue and red curves, respectively) together with the static scattering from ethanol (gray curve) and a scaled literature reference for the ethanol solvent response to an impulsive heating (green curve) (Kjaer *et al.*, 2013 ▸). The difference curves collected in solution agree well with the literature reference for the solvent, while still containing additional signal contributions from the laser-induced structural change in the solute, which are visible especially in the *q* ranges below 1 Å^−1^ and above 3.5 Å^−1^. Each difference curve is obtained after ∼3 min of measurement and reaches sufficient signal-to-noise ratio, despite the small relative difference signal strength not exceeding 0.2%.

## 8. Conclusions

Here we present a flexible environment chamber dedicated to experiments using liquid jet samples at the FXE Instrument at the European XFEL. The chamber was designed with very large openings on three sides, allowing simultaneous spectroscopic and diffraction/scattering experiments to be carried out simultaneously. It also allows the experimental conditions to easily changed between the two different emission spectrometers from FXE located at either side of the chamber without additional interferences, *e.g.* changes in the laser coupling, the sample jet or atmosphere inside the chamber. An advanced experiment detecting signals from both the dispersive and the scanning spectrometers is possible, depending on the Bragg angles needed for the crystal analyzers and the detectors.

A high-speed liquid jet system using either Rayleigh or flat jets compatible with the sub-MHz operation of the FXE Instrument at EuXFEL was detailed. Our portfolio of commercial and 3D printed nozzles provides jets with thicknesses varying from 25 to 500 µm with excellent chemical compatibility for a large variety of solvents. Further nozzle designs aiming at more stable laminar flows and larger areas on flat sheet jets are also envisioned.

The combination of the large area and flexibility of the FXE sample interaction region, the broad photon energy range of operation, and the MHz operation capabilities of EuXFEL enables additional types of demanding experiments that to date were not feasible on other FELs. For instance, fs-resolved XES experiments on very dilute systems and XES experiments combined with WAXS or XRD without reconfiguration of the setup are now routinely executed at FXE and provide broad opportunities for complete mapping of solute and solvation dynamics during ultrafast photochemical processes.

## Figures and Tables

**Table 1:** Values of minimum sample volume and flow rates used on different jet designs Except for the DoD injection, the resulting jet speed is sufficient for ≤0.564 MHz experiments. DoD jets are currently compatible to operation at 23.5 kHz.

Type	Jet thickness (µm)	Volume (ml)	Flow rate (ml min^−1^)
Cylindrical	50	25	6.6
Cylindrical	100	25	26.6
Rectangular	30 × 100	25	10.2
GDVN†	10	5	0.1
DoD	80	<1	0.001

† With orifice diameters of liquid and gas equal to 150 µm and 100 µm, respectively, distance between liquid and gas orifices of 150 µm and applied helium mass flow rate of 25 mg min^−1^.

## Footnotes

1 A solvent compatibility study of IP-S (the most commonly used photopolymer for the Nanoscribe two-photon polymerization printer) was conducted to investigate how well the polyacrylate material can cope with different organic solvents for prolonged exposure times. Three nozzles from the same printing batch were left inside bottles filled with solvents commonly used in experiments at FXE (acetone, acetonitrile, chloroform, dichloromethane, dimethylformamide, dimethyl sulfoxide, tetrahydrofuran, toluene) for 1, 4 and 24 h. After each time point the soaked nozzle was removed from the solvent and cleaned with fresh 2-propanol. After 5 min the nozzles were left to dry for 30 min and finally inspected in an optical microscope and pinched carefully with tweezers to evaluate the degree of swelling and/or damage. IP-S was found to be entirely unaltered after a 24 h bath in each of the solvents. Furthermore, the performance of the nozzle was verified by jetting water under the same conditions as an untreated nozzle for 5 min.
